# Effect of Subgingival Instrumentation on Neutrophil Elastase and C-Reactive Protein in Grade B and C Periodontitis: Exploratory Analysis of a Prospective Cohort Study

**DOI:** 10.3390/jcm11113189

**Published:** 2022-06-02

**Authors:** Peter Eickholz, Anne Asendorf, Mario Schröder, Beate Schacher, Gerhard M. Oremek, Ralf Schubert, Martin Wohlfeil, Otto Zuhr

**Affiliations:** 1 Center for Dentistry and Oral Medicine (Carolinum), Department of Periodontology, Johann Wolfgang Goethe-University Frankfurt/Main, Theodor-Stern-Kai 7, 60596 Frankfurt, Germany; anne.asendorf@googlemail.com (A.A.); m.schroeder@med.uni-frankfurt.de (M.S.); schacher@em.uni-frankfurt.de (B.S.); martinwohlfeil@gmail.com (M.W.); o.zuhr@huerzelerzuhr.com (O.Z.); 2 Centre for Internal Medicine, Department of Laboratory Medicine, Hospital of the Johann Wolfgang Goethe-University Frankfurt/Main, Theodor-Stern-Kai 7, 60590 Frankfurt, Germany; gerhardmaximilian.oremek@kgu.de; 3 Department for Children and Adolescence, Division for Allergy, Pneumology and Cystic Fibrosis, Goethe-University, 60590 Frankfurt, Germany; ralf.schubert@kgu.de; 4 Private Practice Hürzeler/Zuhr, Rosenkavalierplatz 18, 81925 Munich, Germany

**Keywords:** neutrophil elastase, CRP, cytokine(s), non-surgical periodontal therapy, periodontal–systemic disease interactions, periodontitis

## Abstract

Background: Assessment of the effect of subgingival instrumentation (SI) on systemic inflammation in periodontitis grades B (BP) and C (CP). Methods: In this prospective cohort study, eight BP and 46 CP patients received SI. Data were collected prior to and 12 weeks after SI. Blood was sampled prior to, one day, 6, and 12 weeks after SI. Neutrophil elastase (NE), C-reactive protein (CRP), leukocyte count, lipopolysaccharide binding protein, interleukin 6 (IL-6) and IL-8 were assessed. Results: Both groups showed significant clinical improvement. NE was lower in BP than CP at baseline and 1 day after SI, while CRP was lower in BP than CP at baseline (*p* < 0.05). NE and CRP had a peak 1 day after SI (*p* < 0.05). Between-subjects effects due to CP (*p* = 0.042) and PISA (*p* = 0.005) occurred. Within-subjects NE change was confirmed and modulated by grade (*p* = 0.017), smoking (*p* = 0.029), number of teeth (*p* = 0.033), and PISA (*p* = 0.002). For CRP between-subjects effects due to BMI (*p* = 0.008) were seen. Within-subjects PISA modulated the change of CRP over time (*p* = 0.017). Conclusions: In untreated CP, NE and CRP were higher than in BP. SI results in better PPD and PISA reduction in BP than CP. Trial registration: Deutsches Register Klinischer Studien DRKS00026952 28 October 2021 registered retrospectively.

## 1. Introduction

The 1999 Classification of Periodontal Diseases distinguished between chronic (ChP) and aggressive periodontitis (AgP) with regard to onset of the disease at different ages and progressions at different speeds in different patients [1]. Individual predisposition and modifying factors explain these differences. The 2018 classification assigns different grades to different rates of progression (slow progression: (A); moderate progression: (B); rapid progression: (C)). Molar–incisor pattern and case phenotype are elements of the actual classification that recollect AgP [2].

Tooth brushing, flossing, and even chewing may frequently cause bacteraemia in patients with severe untreated periodontitis [3]. AgP and periodontitis grade C exhibit more rapid progression. This may be due to a hyperinflammatory phenotype that also may result in a higher systemic inflammatory burden (i.e., serum C-reactive protein (CRP) and neutrophil elastase (NE)), as shown for AgP [4,5,6,7]. Frequent bacteraemia and the systemic spill of proinflammatory cytokines [4] from periodontal pockets result in the release of NE and acute-phase proteins (e.g., CRP). NE and CRP are markers of systemic inflammatory burden and may be part of the link that connects the oral inflammation periodontitis with other parts of the body. Non-surgical periodontal therapy (subgingival instrumentation: SI) resulted in a serum NE reduction in AgP but not in ChP [5]. Is this difference still detectable if patients are reclassified according to the 2018 classification?

This is an exploratory analysis of a prospective cohort study that originally observed serum NE, CRP, and LPS binding protein to be significantly higher in AgP than ChP and a significant difference regarding the change of serum NE 12 weeks after SI between AgP and ChP [4,5]. Therefore, the primary aim of this exploratory analysis was to compare these inflammatory serum parameters at baseline and after non-surgical subgingival instrumentation (SI) in the same patients after reclassification according to the 2018 classification into grade B (BP) and C (CP) periodontitis.

## 2. Material and Methods

This is the exploratory analysis of data of a prospective cohort study on the effect of SI on serum inflammatory parameters. An exploratory analysis of haematological parameters and heat shock protein 27 has been published recently [7]. Clinical examinations and therapy have been described in detail before [5,7]. Thus, only a brief description is provided in the following. Sixty-six patients with untreated severe periodontal disease (31 generalised severe ChP; 35 AgP) were recruited at the Department of Periodontology of the Center for Dentistry and Oral Medicine (Carolinum), Johann Wolfgang Goethe-University Frankfurt/Main, Germany.

The following inclusion criteria had to be fulfilled: (1) at least 16 years of age, (2) at least 20 remaining teeth and (3) written informed consent.

Patients were diagnosed as aggressive periodontitis if the following criteria were present: (1) clinically healthy patients, i.e., he or she does not suffer from systemic diseases predisposing to periodontitis (e.g., diabetes mellitus); (2) probing pocket depths (PPD) ≥ 3.6 mm at more than 30% of sites [5] (according to the Periodontal Screening and Recording (PSR) index [8] and the directives for treatment of statutorily insured patients in Germany [9], a PPD of 3.5 mm was the threshold for periodontal disease and thus requirement of therapy. However, Florida probes provide measurements to the nearest 0.2 mm. Thus, the threshold for treatment requirement was PPD ≥ 3.6 mm); (3) radiographic bone loss of at least 50% at a minimum of 2 separate teeth; (4) age at time of diagnosis up to 35 years (severe periodontitis below age up to 35 years is a rough threshold to identify rapid destruction in AgP) [4,10]; (5) age at time of recruitment up to 37 years of age [4].

Patients were diagnosed as generalised severe chronic periodontitis if the following criteria were fulfilled: (1) PPD at least 3.6 mm and probing vertical attachment loss (PAL-V) at least 5 mm at more than 30% of sites, (2) PPD at least 7 mm at a minimum of 4 sites (to provide a minimum of deep pockets in each patient); (2) older than 35 years of age.

The following inclusion criteria led to exclusion: (1) requirement of preventive use of systemic antibiotics for measurements that may cause transitory bacteraemia (e.g., pocket probing); (2) self-reported chronic disease influencing the serum CRP level (e.g., rheumatoid arthritis, Crohn’s disease or ulcerative colitis); (3) self-reported infectious disease within the last 8 weeks before examination (history of fever); (4) any clinically assessed chronic dermal or mucosal inflammatory condition (e.g., lichen planus); (5) non-surgical or surgical periodontal treatment within the last 24 months before examination; (6) systemic or topical subgingival antibiotics within the last 8 weeks before examination.

The following parameters were assessed as self-report: (1) current body weight and height, (2) current and past cigarette smoking habits. Patients currently smoking or having quit smoking for less than five years were classified as smokers [11]. Additionally, ethnic origin was recorded [4]. The study protocol fulfilled the rules of the Declaration of Helsinki. The Institutional Review Board for Human Studies of the Medical Faculty of the Goethe-University Frankfurt/Main approved the protocol (Application# 188/06). Information on the risks and benefits as well as the procedures of the study was provided to all participants.

### 2.1. Clinical Examination

An earlier publication of our group reports clinical examinations in detail [4].

One experienced examiner (MW) performed all measurements. He assessed the following parameters at 6 sites per tooth (mesiobuccal, buccal, distobuccal, mesiooral, oral, distooral) at baseline (T0), 6 (T2), and 12 weeks (T3) after SI: (1) Gingival Bleeding Index (GBI) [12], (2) Plaque Control Record (PCR) [13]. MW scored probing parameters immediately prior to the first session of SI and at T3. With an electronic probe (Florida Probe, Version 3.2, Gainesville, FL, USA), he assessed PPD (standard probe) and relative vertical probing attachment level (RAL-V) (disk probe) to the nearest 0.2 mm. Thirty seconds after probing, he scored bleeding on probing (BOP). MW assessed recession to the nearest 0.5 mm using a manual periodontal probe (PCPUNC 15, Hu-Friedy, Chicago, IL, USA) from the cemento-enamel junction (CEJ) to the gingival margin and calculated PAL-V as the sum of PPD and recession. If CEJ was located apical to the gingival margin, PAL-V was calculated as PPD minus the distance from the gingival margin to the CEJ.

### 2.2. Reclassification

Assignment of stage for each patient was performed using the baseline interproximal PAL scores and number of teeth lost [2]. Due to the fact that only patients suffering from AgP or generalised severe ChP had been included originally, it was assumed that all missing teeth had been lost due to periodontitis with the exception of missing 3rd molars that were never considered as lost due to periodontal reasons. For each patient, the percentage of teeth indicating stage III (CAL-V ≥ 5 mm, PPD ≥ 6 mm, furcation involvement class II and III) was documented [7].

Each patient was assigned to a grade using radiographs obtained at baseline (primary criteria) as well as modifying factors (smoking, diabetes mellitus). An experienced periodontologist (PE) viewed the radiographs on a screen (Universial Viewer, Dentsply Rinn^®^, New York, NY, USA) in a darkened room. At the tooth with most severe bone loss, the distances from the CEJ to the most apical extension of bone loss (BD) and to the tip of the root were measured to the next 1.0 mm with a periodontal probe (PCPUNC15, HuFriedy, Chicago, IL, USA). The distance from CEJ to BD was divided by the distance CEJ to root tip to calculate bone loss relative to root length. The division of relative bone loss by patients’ age provided the bone loss age coefficient [7]. Patients with bone loss age coefficient >1 were assigned to grade C [2].

### 2.3. Blood Samples

Twenty millilitres of blood was sampled from an arm vein (T0; T1: one day later immediately prior to the 2nd session of SI; T2, T3). Serum levels of CRP, NE and leukocyte count were analysed at the Department of Laboratory Medicine of the Centre for Internal Medicine, Hospital of the Johann Wolfgang Goethe-University Frankfurt/Main.

Serum IL-6, IL-8, and lipopolysaccharide-binding protein (LBP) concentrations were analysed in duplicates by the ELISA technique according to the manufacturers’ instructions at the Department for Children and Adolescence, Division for Allergy, Pneumology and Cystic Fibrosis, Goethe-University, Frankfurt, Germany. All laboratory methods have been described in detail before [5].

### 2.4. Anti-Infective Therapy

All patients underwent oral hygiene instructions and professional prophylaxis until the full mouth plaque score (PCR) was ≤50% (1st step of therapy) [14]. SI was performed in 2 visits on 2 consecutive days under local anaesthesia (UDS, Sanofi-Aventis Deutschland GmbH, Frankfurt/Main, Germany) according to a modification of the full-mouth disinfection protocol [5,15] (2nd step of therapy). All teeth exhibiting PPD ≥ 3.5 mm underwent SI using sonic scalers (Sonicsys, KaVo, Biberach, Germany) and hand instruments. If *A. actinomycetemcomitans* had been detected from subgingival plaque, 500 mg amoxicillin and 400 mg metronidazole were prescribed 3 times daily for 7 days. In case of sensitivity to penicillin, 250 mg ciprofloxacin and 500 mg metronidazole were prescribed 2 times daily for 7 days [16,17,18]. For 14 days after start of SI for all patients, oral home care included rinsing 2 times daily for 60 s with 10 mL of 0.12% chlorhexidine mouth wash (ParoEx, Sunstar, Schönau, Germany), which was followed by tooth brushing and brushing the back of the tongue with 1% CHX gel. At T2 and T3, all patients received oral hygiene instructions and professional prophylaxis [7].

### 2.5. Statistical Analysis

Statistical analysis was performed using a PC program (Systat^TM^ for Windows Version 13, Systat Inc., Evanston, IL, USA). The sample size had originally been calculated for the main outcome variables NE and CRP for a comparison between ChP and AgP [6,8]. Inferential statistics were intended to be exploratory, not confirmatory. *p*-values represent a metric measure of evidence against the respective null hypothesis and were used only to generate new hypotheses. Therefore, no adjustment for multiple testing was applied. *p*-values < 0.05 were considered as significant. For a description of demographic and clinical parameters, standard univariate statistical analyses were performed. Numbers and percentages describe categorical variables. Continuous variables are reported as means and standard deviations for clinical parameters and as medians (lower/upper quartile) for serum parameters. Patients’ characteristics were compared between BP and CP patients as well as between patients treated with adjunctive systemic antibiotics or not using Fisher’s exact tests for categorical variables and Mann–Whitney U tests for continuous data.

For all individuals, the body mass index (BMI) and cigarette pack years were calculated. Group frequencies (BP, CP) were expressed for sex and current smoking. Group means and standard deviations were calculated for the following parameters: age, number of remaining teeth, pack years, BMI, GBI, PCR and BOP at baseline and 12 weeks as well as for the changes between baseline and 12 weeks. For all site-based periodontal parameters (PPD, PAL-V, RAL-V), means per individual were calculated at T0 and T3 as well as for changes from T0 to T3. Furthermore, using these numbers, group means and standard deviations were calculated. Additionally, the periodontal inflamed surface area (PISA) was calculated per individual to describe the size of the interface between the periodontal pocket and vascular system [19].

For comparisons, repeated measures analysis of variance (MANOVA) was used for log-transformed NE and CRP with the following independent variables: time point of examination (T0, 1, 2, 3), diagnosis (BP = 0, CP = 1), African origin, female sex, smoking (never and former smoker = 0, current smoker = 1), adjunctive systemic antibiotics (no = 0, yes = 1), number of teeth, BOP, BMI and baseline PISA. An effect with a probability of *p* < 0.05 was accepted as significant. 

## 3. Results

Between October 2006 and December 2009, 31 ChP and 29 AgP patients were enrolled. Of originally 56patients three had been recruited but were not enrolled because they violated the inclusion criteria. Furthermore, three patients did not attend the baseline examination and were also not enrolled. The results on these 60 patients’ NE, CRP, leukocyte counts, IL-6 and IL-8, as well as LBP regarding ChP and AgP had already been published [5]. Because the respective radiographs were not available anymore, the assignment of new diagnoses according to the 2018 classification was not possible in six of 60 patients. Thus, the data of 54 patients were analysed [7]. Of those patients assigned to grade B according to interproximal bone loss (%) divided by age, neither was a current heavy smoker (≥10 cigarettes per day) nor suffered from diabetes mellitus. Thus, modifying factors did not affect grade. A total of originally 24 AgP were reclassified to 19 generalised stage III (all CP), none in stage IV and 5 molar incisor pattern (all CP). A total of originally 30 ChP were reclassified to 25 generalised stage III (5 BP, 20 CP), 5 stage IV (3 BP, 2 CP) and no molar incisor patterns [7]. One BP (12.5%) and 24 CP (52.2%) were female (*p* = 0.056), all BP and 39 CP (84.8%) were of European ethnicity, two CP (4.3%) were of African and 5 (10.9%) of Asian ethnicity (*p* = 0.497). The BP group consisted of one current (12.5%) and two (25%) former smokers (CP: current: 15/31% (*p* = 0.411); former: 9/20% (*p* = 0.659)). Four BP (50%) and 17 CP (37%) received SI with adjunctive systemic antibiotics (*p* = 0.697). Table 1 provides further patient characteristics.

After SI, clinical parameters (GBI, BOP, PPD, RAL-V, PISA) improved in general significantly (*p* < 0.05). Only PCR improvement at T3 in BP was not significant. At T3, PPD (*p* = 0.006) and PISA (*p* = 0.046) were significantly better in BP than in CP (Table 2) [7]. Adjunctive systemic antibiotics (AB) failed to make a difference with regard to PPD reduction (AB: −1.3 ± 0.5) mm; no AB: −1.0 ± 0.4 mm; *p* = 0.186) and CAL gain (AB: 0.6 ± 0.4 mm; no AB: 0.4 ± 0.3 mm; *p* = 0.059).

CP exhibited higher serum NE than BP at all time points. However, this difference was significant only at T0 and T1. Furthermore, NE was significantly increased at T1 (*p* < 0.05) (Table 3). Repeated measures analysis of variance identified between subjects significant effects due to CP (*p* = 0.042) and PISA (*p* = 0.005). Within subjects, the change of serum NE over time was confirmed and modulated by grade (*p* = 0.017), smoking (*p* = 0.029), number of teeth (*p* = 0.033), and PISA (*p* = 0.002). Adjunctive systemic antibiotics failed to modulate change of serum NE (Table 4).

Serum CRP was only significantly higher in CP than in BP at T0 (*p* = 0.033). Furthermore, the percentage of patients with serum CRP < 0.1 mg/dl was significantly lower in CP than in BP (*p* = 0.015) (Table 3). Repeated measures analysis of variance identified between subjects significant effects due to BMI (*p* = 0.008). Within subjects, the change of serum CRP over time was modulated by PISA (*p* = 0.017). Adjunctive systemic antibiotics failed to modulate the change of serum CRP (Table 5).

Univariate analysis failed to find any significant difference between BP and CP for leucocyte count, serum LBP, IL-6, and IL-8. However, LBP was significantly increased at T1 for CP (*p* < 0.001) and IL-6 for BP and CP (*p* < 0.05) (Table 6).

## 4. Discussion

This is an exploratory analysis of a prospective cohort study that originally observed serum NE, CRP, and LPS binding protein to be significantly higher in AgP than ChP and observed a significant difference regarding change of serum NE 12 weeks after SI (T3) between AgP and ChP [4,5]. The primary aim of this exploratory analysis therefore was to compare these inflammatory serum parameters at T0 and after step 2 periodontal therapy (SI) [14] in the same patients after reclassification according to the 2018 classification into eight patients with untreated grade B (BP) and 46 with grade C (CP) periodontitis (6 patients were lost due to missing data). In both groups, significant clinical improvement was achieved (*p* < 0.05). NE was significantly lower in BP than CP at T0 and T1, while CRP was significantly lower in BP than CP only at T0. NE and CP were significantly higher at T1 than at T0, 2 and 3. Between-subjects significant effects due to CP and PISA were observed. Change of NE over time was modulated by grade, smoking, number of teeth, and PISA, and significant effects due to BMI were seen. Change of serum CRP over time was modulated by PISA. In untreated CP, serum NE and CRP are higher than in BP. SI results in better PPD and PISA 12 weeks after SI in BP than CP. Adjunctive systemic antibiotics modulated neither change of serum NE nor of CRP.

Oral microbiota may enter internal tissues and circulation via the parakeratinised and ulcerated pocket epithelium of established gingivitis and periodontitis. Summing up the pocket walls of all periodontally compromised teeth in an untreated patient, the periodontal wound surface is estimated to be as large as 8 to 20 cm^2^ [20]. The size of this wound surface was assessed in this analysis as PISA which ranged in this study from 9 to 15 cm^2^ in BP and from 3 to 25 cm^2^ in CP at T0.

Bacteraemia from periodontal pockets and the resulting systemic spill of proinflammatory cytokines cause an acute inflammatory host response [21,22,23]. The cohort studied in this analysis exhibited in AgP significantly higher serum NE and CRP at baseline and 12 weeks after treatment than in ChP [5]. This significant difference persisted even 5 years after treatment, indicating a stronger inflammatory response in AgP than in ChP [6]. In the 1999 classification, AgP represents periodontitis with rapid progression, whereas in the 2018 classification, rapid progression is classified by grade C. With serum NE being significantly higher in CP than BP at baseline and 1 day after SI and CRP at baseline, this exploratory analysis confirms the observation made in AgP for CP. One day after SI, an inflammatory host response was observed in the patients of this study as elevated levels of NE, CRP, LBP, and IL-6 in both ChP and AgP [5]. In this exploratory analysis, the same was observed. Elevation of NE, CRP, and IL-6 was significant for CP and BP. However, for LBP, this was observed only for CP. This difference may be due to the small size of the BP group (*n* = 8).

This study failed to provide any significant differences between BP and CP with regard to leukocyte count, LBP, IL-6 and IL-8. This confirms the results comparing ChP and AgP for leukocyte counts, IL-6 and IL-8. However, for LBP, higher serum LBP was observed in AgP at baseline and 1 day after SI than ChP. This difference may also be due to the small size of the BP group (*n* = 8).

Untreated periodontitis associated with elevated serum NE and CRP may thereby contribute to the risk for CVD and COPD. For AgP, serum CRP was reduced by 0.23 mg/dL at T3 [5]. This was far more than the weighted mean (0.067 mg/dL) calculated by a structured review over two studies which included a total of 40 patients with generalised severe periodontitis [24]. However, neither difference reached statistical significance. Interestingly, in contrast to the analysis of ChP versus AgP, this exploratory analysis failed to show SI reducing any of the investigated systemic inflammatory parameters. In part, this may be due to the loss of patients and rearrangement of groups.

Non-surgical periodontal therapy in this study was effective. It resulted in significant mean PPD reduction (BP: 1.2 mm; CP: 1.0 mm) and attachment gain (BP: 0.4 mm; CP: 0.5 mm), confirming results reported by other groups [25,26,27].

What are the limitations of this analysis? First of all, this is an exploratory analysis of a cohort originally aiming at serum NE and CRP in comparison of ChP and AgP [5]. Since this distinction has been abandoned in the 2018 classification of periodontal diseases, this is an attempt to use the new diagnoses (BP/CP). The exploratory analyses were not adjusted for multiple testing. Thus, there is a high risk to detect differences that are due to chance. Furthermore, the sample size is quite small with a high risk of being underpowered. Another weakness is connected to staging according to tooth loss due to periodontitis. Most patients cannot name the exact reason why teeth have been extracted in particular if tooth loss is a while ago. If extractions have not been performed in one’s own clinic, it is quite difficult to estimate the reason. This is a general difficulty of this parameter in the 2018 classification. Due to the fact that only patients suffering from AgP or generalised severe ChP had been included originally, it was assumed that all missing teeth had been lost due to periodontitis with the exception of missing 3rd molars that were never considered as lost due to periodontal reasons. This may be a pragmatic approach to stage periodontitis due to tooth loss in this analysis. However, to the best of our knowledge, this is the first analysis to compare BP and CP regarding systemic inflammatory host responses.

## 5. Conclusions

Within the limitations of the present study, the following conclusion may be drawn: In untreated grade C periodontitis (CP), serum NE and CRP are higher than in grade B periodontitis (BP). SI results in better PPD and PISA reduction in BP than CP.

## Figures and Tables

**Table 1:** Individuals’ characteristics.

Parameters Mean ± Standard Deviation	Periodontitis Grade B (*n* = 8)	Periodontitis Grade C (*n* = 46)	Grade B/C *p*
Age (years)	61.0 ± 6.7	40.5 ± 11.5	<0.001
Remaining teeth (*n*)	26.6 ± 3.2	27.4 ± 2.9	0.492
Pack years	6.8 ± 11.6	8.4 ± 15.0	0.665
Body mass index (kg/m^2^)	24.4 ± 2.8	26.2 ± 4.7	0.324

**Table 2:** Individuals’ periodontal variables and change of periodontal variables after therapy (PAL-V: clinical vertical attachment level; RAL-V: relative vertical attachment level).

Parameters Mean ± Standard Deviation		Periodontitis Grade B (*n* = 8)	Periodontitis Grade C (*n* = 46)	Grade B/C *p*
Gingival Bleeding Index (%)	Baseline	15.0 ± 7.9	13.4 ± 10.8	0.526
	6 weeks	6.3 ± 5.3 ^a^	3.7 ± 4.7 ^b^	0.113
	12 weeks	6.4 ± 6.5 ^a^	5.9 ± 4.8 ^b,c^	0.981
Plaque Control Record (%)	Baseline	40.1 ± 26.2	35.8 ± 14.1	0.856
	6 weeks	22.0 ± 14.5 ^a^	31.5 ± 18.6 ^a^	0.169
	12 weeks	32.8 ± 11.7	28.2 ± 16.8 ^a^	0.233
Bleeding on probing (%)	Baseline	49.4 ± 15.9	52.0 ± 13.7	0.575
	12 weeks	20.3 ± 7.6 ^a^	26.7 ± 10.4 ^b^	0.092
Probing pocket depth (PPD) (mm)	Baseline	3.4 ± 0.3	3.8 ± 0.7	0.242
	12 weeks	2.2 ± 0.3 ^a^	2.6 ± 0.4 ^b^	0.006
PPD reduction (mm)		1.2 ± 0.3	1.1 ± 0.5	0.342
Attachment level (mm) (PAL-V)	Baseline	4.3 ± 1.0	3.6 ± 1.8	0.093
(RAL-V)	Baseline	11.1 ± 1.2	10.8 ± 1.5	0.715
(RAL-V)	12 weeks	10.6 ± 1.2 ^a^	10.4 ± 1.4 ^b^	0.715
Attachment gain (mm) (ΔRAL-V)		0.4 ± 0.3	0.5 ± 0.4	0.789
PISA (mm^2^)	Baseline	1169 ± 201	1372 ± 543	0.273
	12 weeks	288 ± 112 ^a^	460 ± 303 ^b^	0.046

Significantly different to baseline ^a^ (*p* < 0.05); ^b^ (*p* < 0.001). Significantly different to 6 weeks ^c^ (*p* < 0.05).

**Table 3:** Individuals’ neutrophil elastase and C-reactive protein (median (lower/upper quartile)).

Parameters		Periodontitis Grade B (*n* = 8)	Periodontitis Grade C (*n* = 46)	Grade B/C *p*
Neutrophil elastase (ng/mL) (NE)	Baseline	8.75 (7/13.25)	30.55 (12.4/37.2)	0.008
	1 day	19.3 (14.5/25.1) ^b^	33.05 (21.8/40.1) ^a^	0.036
	6 weeks	9.4 (8.01/17.55)	32 (13.1/37.68)	0.059
	12 weeks	17.65 (10.75/34.65)	28 (11.3/36.2)	0.422
Change baseline to 12 weeks		3.75 (−0.14/0.05)	−1.15 (−4.2/1.4)	0.051
C-reactive protein (mg/dL) (CRP)	Baseline	0.09 (0.08/0.13)	0.17 (0.10/0.34)	0.033
	1 day	0.75 (0.23/1.41) ^a^	0.54 (0.29/1.16) ^a^	0.990
	6 weeks	0.15 (0.05/0.27)	0.17 (0.11/0.34)	0.336
	12 weeks	0.14 (0.07/0.21)	0.23 (0.10/0.34)	0.278
Change baseline to 12 weeks		0.01 (−0.05/0.14)	0 (−0.06/0.05)	0.542
CRP reduction ≥ 0.3 mg/dL (*n*/%) baseline to 12 weeks		0 (0)	4 (9)	1.000
CRP < 0.1 mg/dL (*n*) (%)	Baseline	5 (63)	8 (17)	0.015
	12 weeks	3 (37)	11 (24)	0.413
CRP 0.1 to 0.3 mg/dL (*n*) (%)	Baseline	3 (37)	26 (57)	0.449
	12 weeks	4 (50)	20 (43)	1.000
CRP > 0.3 mg/dL (*n*) (%)	Baseline	0 (0)	12 (26)	0.176
	12 weeks	1 (13)	15 (33)	0.411

Significantly different to all other time points ^a^ (*p* < 0.001), ^b^ (*p* < 0.05).

**Table 4:** Repeated measures analysis of variance of log-transformed neutrophil elastase (NE).

	Degrees of Freedom	F-Ratio	*p*-Value
Between subjects			
Grade C	1	4.397	0.042
African origin	1	2.589	0.115
Female	1	0.464	0.499
Bleeding on probing (T0)	1	1.669	0.203
Smoker	1	1.359	0.250
Systemic antibiotics	1	0.001	0.973
Number of teeth	1	1.079	0.305
Body mass index (T0)	1	2.716	0.107
PISA (T0)	1	8.858	0.005
Error	43		
Within subjects			
NE	3	3.406	0.020
NE × grade C	3	3.533	0.017
NE × African origin	3	0.666	0.574
NE × female	3	0.107	0.956
NE × bleeding on probing (T0)	3	0.481	0.696
NE × smoker	3	3.091	0.029
NE × systemic antibiotics	3	1.203	0.312
NE × number of teeth	3	3.005	0.033
NE × body mass index	3	0.524	0.666
NE × PISA (T0)	3	5.218	0.002
Error	129		

**Table 5:** Repeated measures analysis of variance of log-transformed C-reactive protein (CRP).

	Degrees of Freedom	F-Ratio	*p*-Value
Between subjects			
Grade C	1	0.254	0.617
African origin	1	1.385	0.246
Female	1	0.047	0.830
Bleeding on probing (T0)	1	0.358	0.553
Smoker	1	0.004	0.950
Systemic antibiotics	1	1.284	0.263
Number of teeth	1	0.178	0.675
Body mass index (T0)	1	7.691	0.008
PISA (T0)	1	1.129	0.294
Error	44		
Within subjects			
CRP	3	0.539	0.657
CRP × grade C	3	1.311	0.274
CRP × African origin	3	0.581	0.628
CRP × female	3	0.248	0.863
CRP × bleeding on probing (T0)	3	0.818	0.486
CRP × smoker	3	0.294	0.829
CRP × systemic antibiotics	3	2.238	0.087
CRP × number of teeth	3	0.243	0.866
CRP × body mass index	3	0.467	0.706
CRP × PISA (T0)	3	3.539	0.017
Error	132		

**Table 6:** Leukocyte counts LPS binding protein, interleukin 6 and 8 (median (lower/upper quartile)).

Parameters		Periodontitis Grade B (*n* = 8)	Periodontitis Grade C (*n* = 46)	Grade B/C *p*
Leukocyte count (nL^−1^)	Baseline	5.86 (3.95/7.62)	6.37 (5.12/7.47)	0.559
	1 day	5.05 (4.51/6.66)	6.34 (4.98/7.34)	0.330
	6 weeks	5.01 (4.56/5.79)	6.02 (4.9/7.49) ^b^	0.189
	12 weeks	4.92 (4.29/5.30)	5.97 (4.85/7.5)	0.056
LPS binding protein (µg/mL) (LBP)	Baseline	22.2 (15.3/27.9)	30.4 (22.3/45) ^a^	0.108
	1 day	40.4 (25.5/49.7)	44.3 (31.7/57.6)	0.488
	6 weeks	32 (20.5/43.1)	26.6 (19.5/42.9) ^a^	0.961
	12 weeks	34 (18/44)	24.7 (19.2/36.8) ^a^	0.618
Interleukin 6 (pg/mL) (IL-6)	Baseline	1.55 (1.25/2.5) ^b^	1.5 (0.9/2) ^a^	0.510
	1 day	2.95 (2.25/3.65)	2.8 (2/5.1)	0.855
	6 weeks	1.25 (1/3) ^b^	1.25 (0.8/1.7) ^a,c^	0.626
	12 weeks	1.2 (0.85/2.55) ^a^	1.5 (1.1/2.2) ^a,d^	0.502
Interleukin 8 (pg/mL) (IL-8)	Baseline	21.5 (17/29)	17 (11/25)	0.223
	1 day	27 (24/35.5)	20.5 (13/29)	0.125
	6 weeks	36 (20/58.5)	17 (13/28)	0.108
	12 weeks	24.5 (19.5/29)	22.5 (15/37) ^c^	0.884

Significantly different to 1 day ^a^ (*p* < 0.001); ^b^ (*p* < 0.05); significantly different to baseline ^c^ (*p* < 0.05); significantly different to 6 weeks ^d^ (*p* < 0.05).

## Data Availability

The datasets used and/or analysed during the current study are available from the corresponding author on reasonable request.

## Abbreviations

AgP	aggressive periodontitis
BD	most apical extension of bone loss
BMI	body mass index
BOP	bleeding on probing
BP	grade B periodontitis
CEJ	cemento-enamel junction
ChP	chronic periodontitis
COPD	chronic obstructive pulmonary disease
CP	grade C periodontitis
CRP	C-reactive protein
CVD	cardiovascular disease
GBI	gingival bleeding index
IL	interleukin
LBP	lipopolysaccharide-binding protein
MANOVA	repeated measures analysis of variance
NE	neutrophil elastase
PAL-V	vertical probing attachment loss
PCR	plaque control record
PISA	periodontal inflamed surface area
PMN	polymorphonuclear leukocytes
PPD	probing pocket depth
RAL-V	vertical relative attachment level
SI	subgingival instrumentation
